# Letter to the Editor

**DOI:** 10.1093/icvts/ivag064

**Published:** 2026-02-27

**Authors:** Claus Juergen Preusse

**Affiliations:** Cardiac Surgery, Rheinisch-Westfälische University of Bonn, Medical Faculty, D-53127 Bonn, Germany

A critical response to the publication by F. Settepani et al. is necessary for the following reasons^1^:

1. Since 2016 and 2019, respectively, regarding the 2 references listed below, are “**state of the art**” and therefore the authors should/must have used the “effect size” (confidence limit) calculation for comparison of the three protection methods.^1^^,^^2^^,^^3^
2. The publication does not indicate whether the groups are comparable and representative, regardless of whether a *P* value or a CI is used as a statistical parameter.
3. A serious aspect is the difference in protection methodology, because in the Custodiol group, all hearts were only perfused initially, whereas in the other 2 groups, cardioplegic reperfusion was performed every 20 minutes. This factor is extremely important, as it has a decisive impact on the ischemic stress and effective ischemic time, respectively.
4. It would have been important and meaningful if the authors had presented the confidence intervals for the 3 groups graphically.

It is very unfortunate that, despite the great effort made by the authors, the article has no clinical relevance due to statistical shortcomings.

## Data Availability

Data are available in a repository and can be accessed via a DOI link.
